# The orchestrated interplay between DNA methylation and N6-methyladenosine modification: *status quo* and future perspectives

**DOI:** 10.7717/peerj.20654

**Published:** 2026-01-27

**Authors:** Jie Yang, Jun Liao, Bi-Wen Mo, Yan Gao, Yun-Xiang Chen, Yue Gu, Miao-Miao Liang, Hui-Min Li

**Affiliations:** 1 Guilin Medical University, Guilin, Guangxi, China; 2 The First Affiliated Hospital, Guilin Medical University, Guilin, Guangxi, China; 3 Guangxi Key Laboratory of Metabolic Reprogramming and Intelligent Medical Engineering for Chronic Diseases, The Second Affiliated Hospital of Guilin Medical University, Guilin, Guangxi, China

**Keywords:** DNA methylation, m6A modification, Crosstalk, Bibliometrics

## Abstract

DNA methylation (DNAme) and N6-methyladenosine (m6A) represent key mechanisms in epigenetic and epitranscriptomic regulation, respectively. While DNAme is a well-established modification, m6A has more recently emerged as a central focus of epitranscriptomic research. This review comprehensively explores the dynamic crosstalk between DNAme and m6A, addressing the molecular intricacies and functional consequences of their interplay. A systematic literature search conducted in Web of Science Core Collection identified 972 publications related to both modifications. After rigorous screening, 29 studies directly investigating interactions between DNAme and m6A were included for in-depth analysis. These interactions were systematically classified into six distinct modes: (1) DNAme-mediated regulation of m6A; (2) m6A-dependent modulation of DNAme; (3) indirect interplay mediated by intermediate factors; (4) direct bilateral regulation between DNAme and m6A; (5) cooperative targeting of common downstream genes or biological processes; and (6) co-expression patterns suggestive of functional interplay. This categorization provides a novel conceptual framework that integrates disparate mechanistic insights, highlights under-explored areas, and proposes new hypotheses for future research. By synthesizing and structuring current knowledge, this review serves as a foundational resource for understanding the complex relationship between these two modifications and facilitates the identification of novel regulatory axes in epigenomic and epitranscriptomic research.

## Highlights

- Bibliometric analysis indicates that research on the interplay between DNA methylation and m6A methylation has been gradually increasing in recent years, yet it remains in its infancy.
- Currently, the reported mechanisms of interaction between DNA methylation (DNAme) and m6A methylation are primarily sixfold: (1) DNAme-m6A regulation, where DNAme influences m6A; (2) m6A-DNAme regulation; (3) The interplay of DNAme and m6A via other intermediate molecules; (4) Direct bilateral regulation of DNAme and m6A molecules; (5) Co-regulation of DNAme and m6A on the same target; (6) Co-expression and potential interaction of these two molecules.
- The most actively interacted molecules identified to date include DNMT1, DNMT3A, DNMT3B, METTL3, METTL14, WTAP, FTO, *etc*.
- Potential functional implications of the crosstalk between DNA-me and m6A: (1) Double enhanced/Competitive/Compensation effect. (2) Signal relay and cost-effective large-scale regulation. (3) Signal storage and rapid release.

## Introduction

Epigenetics encompasses the regulation of heritable phenotypic changes without alterations to the DNA sequence, primarily involving mechanisms such as DNA methylation, histone modifications, chromatin remodeling, and non-coding RNAs (Holliday, 2006). In parallel, epitranscriptomics has emerged as a rapidly advancing frontier, investigating post-transcriptional gene regulation mediated by chemical modifications on RNA—including N6-methyladenosine (m6A), 5-methylcytosine (m5C), pseudouridine ( $\Psi$), and others (Seo & Kleiner, 2021). Both epigenetic and epitranscriptomic mechanisms are recognized as critical interfaces through which environmental signals influence gene expression and cellular phenotypes (Luo et al., 2025; Yang et al., 2025).

Among epigenetic mechanisms, DNA methylation (DNAme) represents one of the most extensively studied modifications, playing fundamental roles in cell differentiation, genomic stability, and transcriptional regulation (Luo et al., 2025). It is catalyzed by DNA methyltransferases (DNMT1, DNMT3A, DNMT3B), interpreted by methyl-binding proteins (MBD1, MBD2, MBD3, MBD4, MeCP2, NEIL1, NTHL1, SMUG1, TDG, UHRF1, UHRF2, UNG, ZBTB33, ZBTB38, ZBTB4), and actively reversed by TET enzymes (TET1, TET2, TET3) (Chen et al., 2020). As a significant pre-transcriptional regulatory mechanism, DNAme disruption can lead to alterations in gene expression levels and the onset of various diseases (Zhang, Lu & Chang, 2020).

On the other hand, m6A methylation—the most abundant internal modification in eukaryotic mRNAs—governs transcript fate through dynamic and reversible regulation mediated by writers (METTL14, METTL3, RBM15, RBM15B, VIRMA, WTAP, ZC3H13), readers (HNRNPA2B1, HNRNPC, IGF2BP1, IGF2BP2, IGF2BP3, RBMX, YTHDC1, YTHDC2, YTHDF1, YTHDF2, and YTHDF3) and erasers (ALKBH5 and FTO), influencing mRNA stability, splicing, translation, and decay (Jiang et al., 2021).

While both DNAme and m6A have been independently scrutinized in diverse biological contexts, emerging evidence underscores their functional interdependence and crosstalk (Li et al., 2024a, 2024b; Wu et al., 2024; Zhang et al., 2024b). For instance, in tomato, DNAme modulates the expression of the m6A demethylase SlALKBH2, which in turn regulates the stability of transcripts encoding DNA demethylases, forming a feedback loop that controls fruit ripening (Zhou, Tian & Qin, 2019). Such reciprocal regulation suggests a layered regulatory architecture that integrates pre- and post-transcriptional control.

However, despite growing interest, no systematic synthesis exists to comprehensively map the modes, mechanisms, and functional outcomes of the interaction between DNAme and m6A methylation. Current literature remains fragmented, often focused on isolated instances rather than overarching principles, and lacks a unified framework that explains how these systems cooperate in development, homeostasis, and disease.

This review aims to fill this critical knowledge gap by synthesizing recent advances into a coherent model of DNAme–m6A crosstalk. We categorize and analyze the molecular mechanisms underlying their interactions, discuss their integrated roles in physiological and pathological processes, and highlight emerging concepts—such as feedback loops, context-specificity, and therapeutic implications—that remain underexplored. By providing a structured overview and proposing future research directions, this work seeks to establish a foundation for understanding complex epitranscriptomic–epigenetic networks and inspire novel interdisciplinary approaches in gene regulation research.

## Methods

### Data sources and search strategy

The Web of Science Core Collection (WOSCC) was selected as the data source due to its comprehensive coverage and high credibility in bibliometric research (Ding & Yang, 2020; Liu et al., 2022). A systematic literature search was conducted on September 21, 2025, using the query: TS = ((“DNA methylation” OR “5-methylcytosine” OR “5mC”) AND (“m6A” OR “N6-methyladenosine” OR “RNA methylation”)), with no restrictions on date, language, or document type. All retrieved records were exported with complete metadata and screened using the Rayyan online platform.

### Study selection process

The selection process adhered to the PRISMA guidelines (Page et al., 2021). Two independent reviewers (J.Y. and J.L.) performed screening after a calibration exercise with 50 sample records, achieving excellent agreement (Cohen’s κ = 0.88). Discrepancies were resolved through discussion or by a third researcher (H.-M.L.).

### Eligibility criteria

Inclusion criteria encompassed original research articles explicitly addressing mechanistic interactions between DNAme and m6A. Exclusions included reviews, meeting abstracts, editorials, letters, retracted publications, and studies focusing solely on one modification without cross-talk analysis.

**Figure 1:**
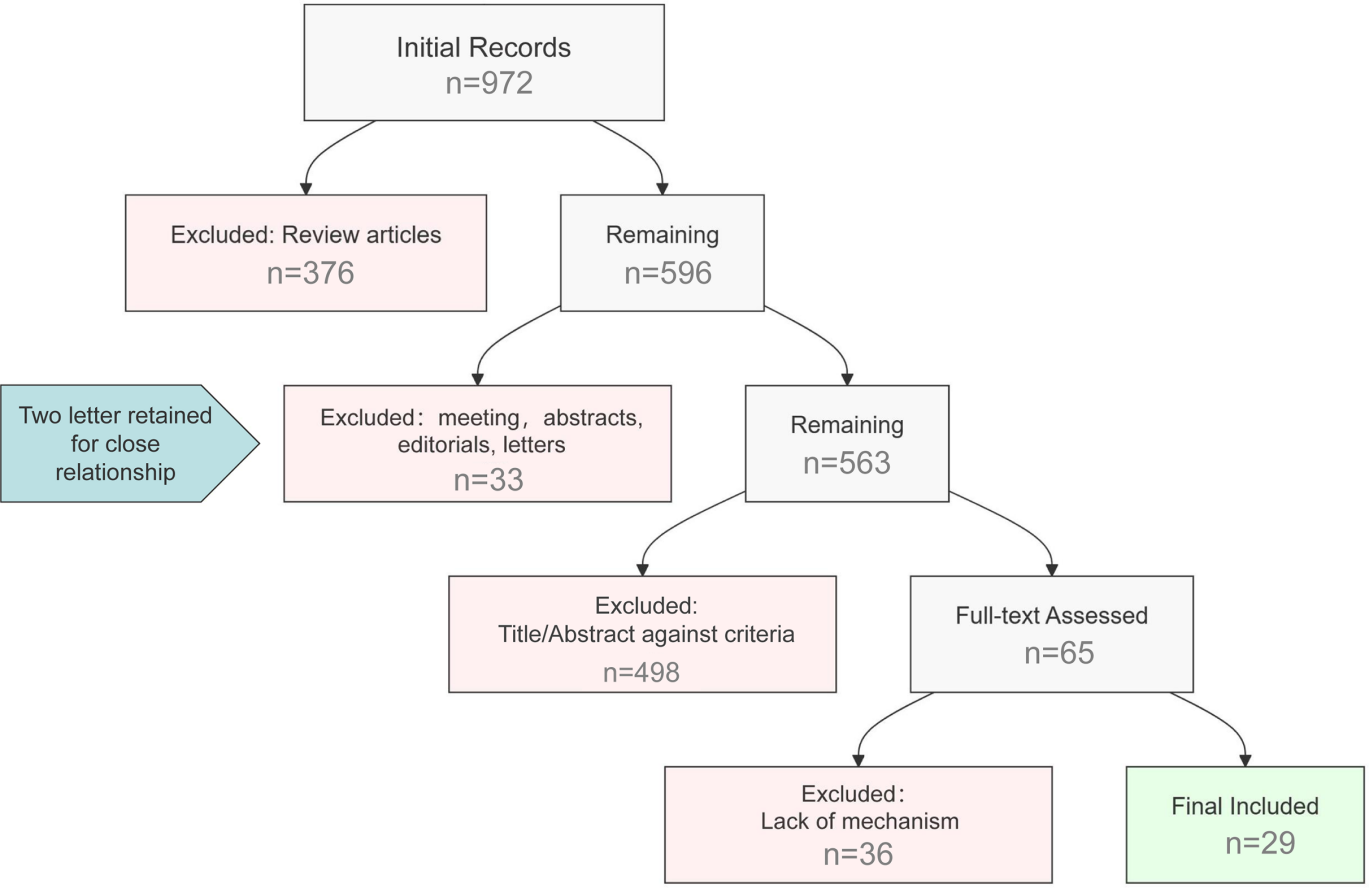
Literature screening flowchart.

The screening process included: 1. Identification of 972 records; 2. Exclusion of 376 reviews, leaving 596 articles; 3. Exclusion of 33 meeting abstracts/editorials (Two relevant letter retained), resulting in 563 records; 4. Title/abstract screening: 498 records excluded, 65 retained; 5. Full-text assessment: 36 articles excluded for lack of mechanistic relevance; 6. Final inclusion: 29 articles. This workflow is summarized in Fig. 1.

### Bibliometric analysis and visualization

Bibliometric metadata were exported from WOSCC and analyzed using R (v4.2.1) with the bibliometrix package (v4.1.2). Key steps included: 1. Data preprocessing: Author Keywords (DE) and Keywords Plus (ID) were merged; synonyms and singular/plural forms were unified manually; 2. Country/Institution analysis: The first author’s affiliation determined the country of publication; 3. Collaboration network: Co-authorship networks were visualized with a threshold of one document per node; 4. Keyword co-occurrence: A network was constructed using a minimum frequency of 2, with links normalized *via* the equivalence index and clusters identified using the walktrap algorithm. Sensitivity analysis (frequency ≥ 3) confirmed cluster stability; 4. Citation & temporal analysis: Journal impact was assessed by total citations; publication trends were analyzed by year. A detailed summary of the characteristics of the included studies is presented in Table S1. The interaction network between DNAme and m6A was constructed using the igraph package within the HiPlot bioinformatics platform (https://hiplot.org).

## Results

### Publication output and bibliometric analysis

**Figure 2:**
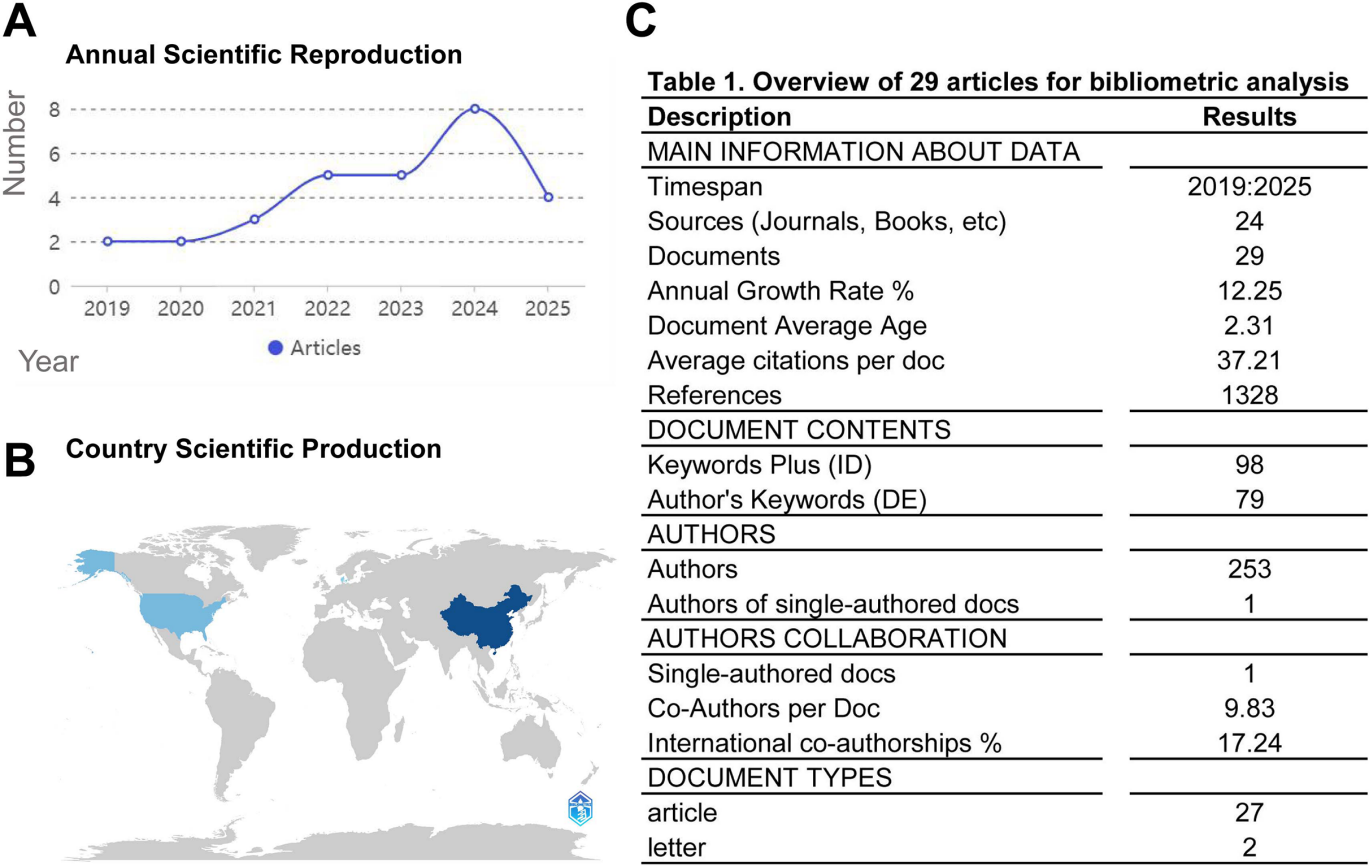
Publication output analysis. (A) Publications production trend. (B) Geographical distribution. (C) Quantitative display of publication output analysis.

To comprehensively characterize the research landscape of interactions between DNAme and m6A, bibliometric analyses were performed on the 29 included articles. Figure 2A illustrates the annual publication trend from 2019 to 2025: starting with two publications in 2019, the number rose steadily to a peak of 8 in 2024, followed by a slight decline to 4 in 2025—noting that data collection for 2025 only covers up to September, and more publications in this research field are likely to be released in the remaining months of the year. This trajectory reflects growing research interest in DNAme–m6A crosstalk. Figure 2B highlights China and the United States as the leading contributors, underscoring their advanced research capabilities and academic focus in this field. Table 1 (integrated within Fig. 2) details key metrics: a 2019–2025 timespan, 24 sources, 29 documents, a 12.25% annual growth rate (indicating rapid field expansion), a 2.31-year average document age, and 37.21 average citations per document (reflecting novel, well-cited research). Diverse keywords (98 Keywords Plus, 79 Author’s Keywords), 253 authors, high collaboration (one single-authored document, 9.83 co-authors per document, 17.24% international co-authorships), and 27 articles + two letters further define the publication landscape.

**Table 1:** The regulation of m6A modification by DNA methylation (Mode 1).

No.	Diseases	DNA methylation	m6A modification	DNAme-m6A regulation	References (PMID)
1	Cardiac hypertrophy	DNMT1	METTL3	Reduced DNMT1 upregulates METTL3.	38314261
2	Pancreatic cancer	DNMT1, DNMT3A	METTL3	Low binding rates of DNMT1 and DNMT3a to METTL3 promoter upregulate METTL3.	31015415
3	Porcine embryo development	TF-SP1 promoter methylation status	METTL14	Inhibiting the DNA methylation level enhances TF-SP1 binding and thereby increases METTL14.	38095020
4	Pig skeletal muscle development	TF-SP1 promoter methylation status	IGF2BP3	Inhibiting the DNA methylation level enhances TF-SP1 binding and thereby increases IGF2BP3.	33434283
5	Intracerebral hemorrhage	cg01637218 site of YTHDF2 promoter	YTHDF2	DNA hypomethylation status of YTHDF2 caused elevated expression YTHDF2	37907028
6	Lung cancer	5mC DNA methylation site of the ALKBH5 promoter	ALKBH5	Hypermethylation of ALKBH5 prevents the binding of the transcriptional repressor CTCF, thereby increase ALKBH5 expression.	34016959
7	Esophageal squamous cell carcinoma	CpG islands of ALKBH5 promoter	ALKBH5	ALKBH5 expression level was increased through the hypomethylation of its promoter.	32169859
8	Alcohol-induced kidney injury	DNMT1, DNMT3A, DNMT3B	FTO	DNMT1, DNMT3A, and DNMT3B can increase the methylation status of FTO, thereby reducing its expression.	33157234
9	Maternal obesity	TETs	WTAP, RBM15B, KIAA1429	The global levels of 5mC levels rose significantly with lowered TET activity, while m6A levels dropped with decreased expression of WTAP, RBM15B, and KIAA1429.	34890771

**Figure 3:**
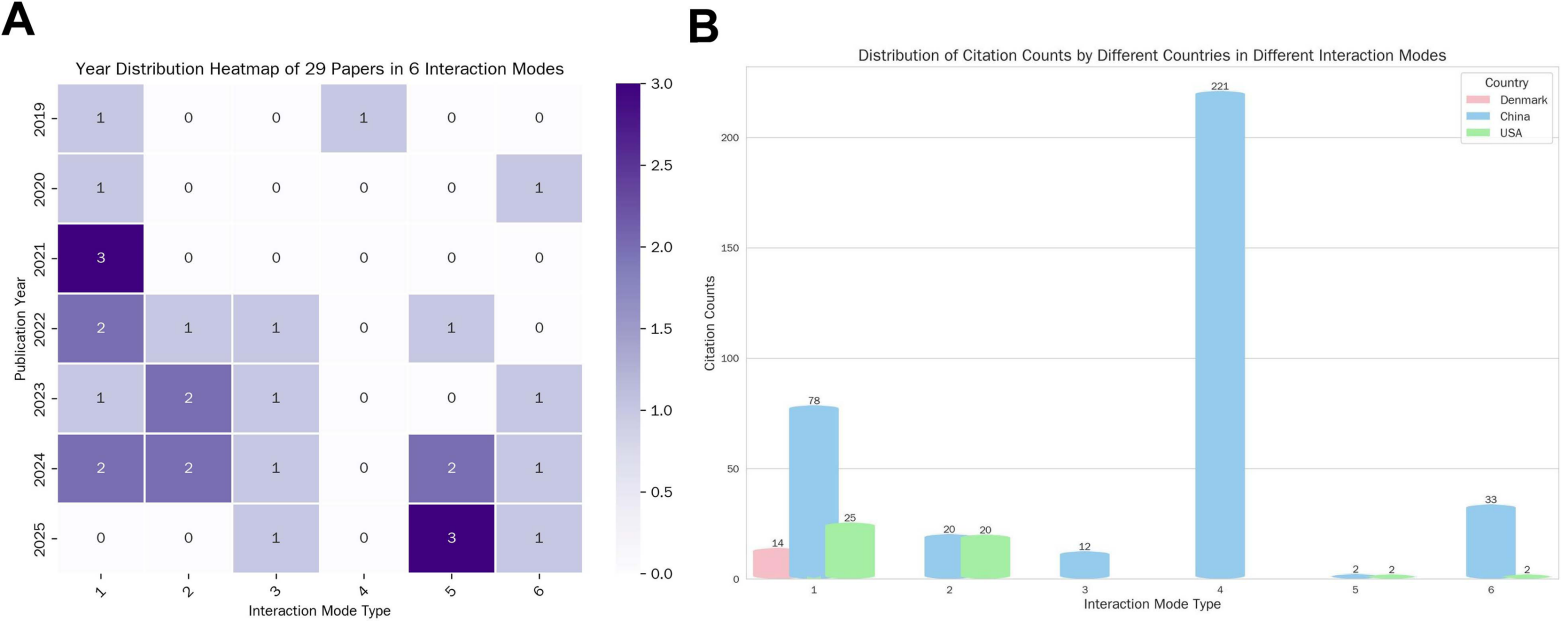
Distribution of publications and citations across DNAme-m6A interaction modes. (A) Heatmap depicting the annual distribution of 29 articles among six defined interaction modes from 2019 to 2025 (color intensity corresponds to publication count per year per mode). (B) Bar chart showing citation counts for each interaction mode by country (China, USA, Denmark), reflecting geographic contributions to high-impact research in this field.

Beyond general trends, analyses of interaction modes revealed nuanced patterns (Fig. 3). Figure 3A shows the yearly distribution of publications across the six defined modes: mode 1 (DNAme-mediated regulation of m6A) and mode 5 (cooperative targeting of common downstream genes/processes) dominated, with 3 publications in 2021 (mode 1) and 2025 (mode 5), respectively. In contrast, modes 3 (indirect interplay *via* intermediates) and 4 (direct bilateral regulation) were minimally represented (mostly 0 publications), indicating uneven exploration of interaction modalities.

Figure 3B delineates the citation counts accrued by publications from China, the USA, and Denmark across each DNAme-m6A interaction mode, with quantitative details supported by the accompanying statistical table. Notably, China emerges as the dominant contributor to citation impact across all six interaction modes: its publications span every mode (*e.g.*, Mode 4, which yielded the field’s highest single-mode citation count of 221; Mode 1, which encompasses multiple high-impact studies), and this output has been sustained consistently over the 2019–2025 period. In contrast, the USA contributes moderately to citation metrics within select modes (specifically Modes 1, 2, 5, and 6), while Denmark’s participation is restricted to Mode 1—with its sole contribution (a 2022 publication) accumulating 14 citations. Collectively, this citation distribution underscores that China is the primary driver of high-impact research in the DNAme-m6A interaction field, whereas the USA and Denmark exhibit narrower participation scopes and smaller citation footprints in this domain.

### Interplay of DNAme and m6A modification

A critical challenge in current DNAme and m6A research lies in two key aspects: first,these two core regulators—DNAme (epigenetic, pre-transcriptional control) and m6A(epitranscriptomic, post-transcriptional control)—have long been studied in isolation,leaving a gap in understanding the integrity of gene regulatory networks. There is a lack of a systematic framework to organize their discrete interaction patterns, which has impeded integrating mechanistic insights, clarifying their synergistic/antagonistic roles in diseases, and guiding dual-target therapeutic strategies. To address this, we propose a novel categorization system for DNAme-m6A crosstalk—based on interaction direction, type, and regulatory logic—that classifies these interactions into six categories (see Graphic Abstract). For the 29 selected studies, we present the number of studies per category as follows: 1. DNAme modulates m6A patterns; nine studies); 2. m6A affects DNAme; five studies; 3. Indirect crosstalk *via* intermediates: four studies; 4. Direct bilateral regulation between DNAme and m6A: one study; 5. Co-regulation of shared genes/biological processes: six studies; 6. Co-expression and potential interaction of the two molecules: four studies.

**Figure 4:**
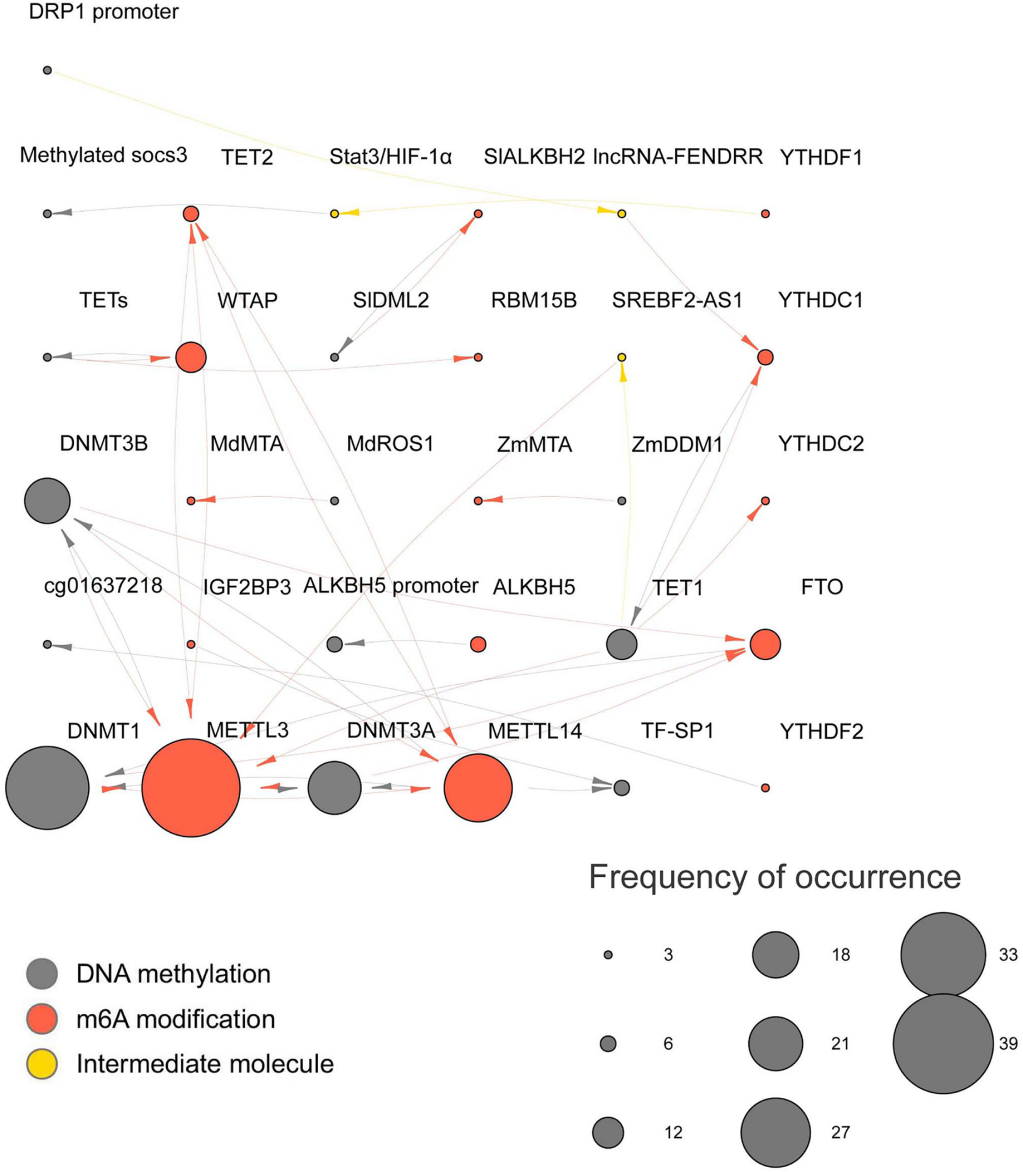
The interaction network of DNA methylation, m6A modification, and intermediary molecules. Gray, red, yellow represent DNA methylation, m6A modification, intermediate molecules respectively. The size of the circle represents the number of occurrences in the interactions. The direction of the arrow indicates the direction of the action.

Concurrently, we compiled all the molecules with interactive relationships and their effects, as depicted in Fig. 4. As identified in the current study, the most active molecules involved in the interplay between DNAme and m6A were DNMT1 and METTL3. Other extensively studied molecules include METTL14, DNMT3A, DNMT3B, WTAP, and FTO. The relationships and directions of these molecular interactions are illustrated in Fig. 4 using arrows.

### DNAme-m6A regulation

Table 1 provides an overview of the published studies on the regulatory effects of DNAme on m6A methylation. DNA methylation occurs in the promoter region of m6A molecule genes. Consequently, this regulation influences the expression and functional status of m6A molecules. For example, in cardiac hypertrophy, reduced DNMT1 levels upregulate METTL3, which in turn regulates cardiomyocyte survival and apoptosis by modulating the expression of ANP/BNP and the m6A modification of apoptosis-related genes; targeting the DNMT1/METTL3 pathway may thus provide a novel therapeutic direction for cardiac hypertrophy (Zhang et al., 2024b). In pancreatic cancer, cigarette smoke condensate induces METTL3 promoter hypomethylation *via* DNMT1/DNMT3A; METTL3 then affects miR-25-3p maturation through direct m6A modification and regulates the AKT-p70S6K pathway *via* PHLPP2, ultimately promoting cancer cell growth (Zhang et al., 2019). During somatic cell nuclear transfer (SCNT) embryo development, DNAme modulates METTL14 expression by interfering with the binding of transcription factor SP1; overexpressing METTL14 increases global m6A levels in donor cells and enhances SCNT embryo developmental efficiency (Zhang et al., 2024a).

The m6A reader proteins are also regulated by DNAme. For example, during pig skeletal muscle development, transcription factor SP1 binds to the IGF2BP3 promoter to regulate IGF2 expression, thereby influencing muscle cell proliferation and differentiation (Yang et al., 2021). In intracerebral hemorrhage, hypomethylation of the YTHDF2 promoter drives high YTHDF2 expression, which alters the stability of functional long non-coding RNAs (lncRNAs) (Zhou, 2023).

For m6A erasers, LKB1 promotes ALKBH5 transcription *via* DNAme modulation, which decreases ALKBH5-mediated m6A demethylation of oncogenes (*e.g*., SOX2, SMAD7, MYC) and increases their mRNA stability/expression, contributing to tumor aggressiveness (Zhang et al., 2021). In esophageal squamous cell carcinoma (ESCC), cigarette smoke condensate induces ALKBH5 promoter hypomethylation to upregulate ALKBH5, which alters the m6A status of LINC00278, influences YY1BM translation, and modulates ESCC cell survival under nutrient deprivation (Wu et al., 2020). During alcoholic cirrhosis, DNMT1/DNMT3A/DNMT3B downregulate FTO, leading to increased m6A modification of PPAR-α, activation of renal inflammatory responses, and exacerbated kidney damage (Yu et al., 2021).

A separate study uncovered a potential global regulatory crosstalk between DNAme and m6A in placental dysfunction: placentas from obese mothers exhibited increased global 5mC levels, reduced TET1/2/3 expression, and decreased m6A levels (with downregulated m6A writers: WTAP, RBM15, KIAA1429) (Shen et al., 2022). The authors proposed a “DNA hypermethylation → transcriptional repression of m6A writers → m6A deficiency” cascade; while direct DNAme-m6A interaction remains unconfirmed, their co-occurrence implies an underlying mechanistic connection to placental dysfunction in maternal obesity.

Collectively, DNAme regulates m6A-related genes primarily by controlling transcriptional initiation, thereby modulating the activity of m6A-dependent signaling networks. Modulating the activity or expression of DNA methyltransferases (*e.g*., DNMT1, DNMT3A) could restore normal promoter methylation status of m6A genes, regulate their expression, and ultimately reset downstream pathway activity. For instance, developing inhibitors targeting DNMT1 and DNMT3A may represent a novel strategy for pancreatic cancer treatment (Zhang et al., 2019).

### The m6A-DNAme regulation

Research on the reverse regulatory effects of m6A modifications on DNAme is also extensive (Table 2), with two primary mechanisms: m6A modulates the expression of DNAme-related enzymes or recruits DNAme regulators to specific genomic loci, thereby reshaping DNA methylation patterns.

**Table 2:** The regulation of DNA Methylation by m6A Modification (Mode 2).

No.	Diseases	m6A modification	DNA methylation	m6A-DNAme regulation	References (PMID)
1	Progression of non-small cell lung cancer	METTL3	DNMT1	METTL3 increased the expression of DNMT1 through m6A methylation.	38586389
2	Esophageal squamous cell carcinoma	METTL3, FXR1	TET1	METTL3-mediated RNA m6A formation facilitates the recruitment of TET1 by FXR1 to achieve TET1’s downstream functions.	36071173
3	Cell fate of human pluripotent stem cells	YTHDC2	TET1	YTHDC2 can recruit TET1 to realize its downstream function.	37474847
4	Apple heterografting	MdMTA (ortholog of METTL3)	MdROS1	Downregulated expression MdMTA induced the global m6A hypomethylation and distinctly activated the expression level of MdROS1.	37648253
5	Maize kernel development	ZmMTA	ZmDDM1	ZmMTA interact with ZmDDM1 and influence the downstream DNA methylation function of ZmDDM1.	37995374

For instance, in non-small cell lung cancer, METTL3 increases DNMT1 expression *via* m6A modification, increasing methylation at the FOXO3a promoter, inhibiting FOXO3a expression, and enhancing tumor cell invasiveness (Li et al., 2024b). In ESCC, METTL3-mediated m6A modification triggers DNA demethylation at adjacent genomic sites through interactions between the m6A reader FXR1 and the DNA 5-methylcytosine dioxygenase TET1. Upon recognizing RNA m6A, FXR1 recruits TET1 to genomic loci for DNA demethylation, altering chromatin accessibility and reprogramming gene transcription. Oncogenes such as WNT7B and BCL6 are transcriptionally activated by increased chromatin accessibility, while tumor suppressors such as FAT4 and SAMD9L are repressed by decreased accessibility, thus promoting ESCC initiation and progression (Deng, Zhang & Su, 2022). In human pluripotent stem cells (hESCs), YTHDC2 recruits the DNA 5-methylcytosine (5mC)-demethylase TET1 to remove 5mC from LTR7/HERV-H, preventing epigenetic silencing and regulating neural differentiation-related gene expression during hESC fate determination (Page et al., 2021).

The interplay between DNA methylation and m6A modifications has also been extensively studied in plants, revealing context-specific regulatory patterns. For instance, during apple grafting, downregulated expression of the m6A methyltransferase gene MdMTA (a METTL3 ortholog) triggers global m6A hypomethylation and significantly upregulates the DNA demethylase gene MdROS1, thereby regulating stress response-related genes (Xu & He, 2023). During corn embryo development, genes with m6A modifications exhibit significantly higher DNA methylation levels compared to those lacking m6A modifications.

Dysfunction of the m6A-modified gene ZmMTA leads to a marked decrease in CHH methylation within the 5′ region of m6A-modified genes, disrupting key biological processes in corn embryo and endosperm development (*e.g*., cell proliferation, differentiation, tissue morphogenesis) (Luo & Guo, 2024). Notably, loss of function of the DNA methylation gene ZmDDM1 does not significantly affect ZmMTA-related activities.

m6A primarily regulates DNA methylation through two pathways: modulating the expression of DNA methylation-related genes *via* m6A modification, or recruiting DNA methylation regulators to downstream gene promoters through m6A-modified molecules. Silencing these m6A-modified genes or disrupting the binding of m6A reader proteins to DNA methylation gene promoters may represent a novel therapeutic strategy. For instance, in ESCC, silencing METTL3 or disrupting FXR1-TET1 binding at downstream oncogene promoters significantly inhibits ESCC cell proliferation, migration, and invasiveness, supporting these targets as potential therapeutic candidates for ESCC (Deng, Zhang & Su, 2022).

### The interplay of DNAme and m6A with intermediate molecules

DNAme and m6A also interact *via* intermediary molecules, a pattern that—while not strict “crosstalk”—reveals their ability to function in a relay manner within the same biological process (Table 3). For example, 5mC-mediated Socs3 repression leads to Stat3 activation, which sequentially activates HIF-1α and downstream YTHDF1; YTHDF1 then regulates collagen-related genes driving liver fibrosis progression (Feng et al., 2023).

**Table 3:** Crosstalk between DNA methylation and m6A modification *via* intermediary molecules (Mode 3).

No.	Diseases	DNA methylation	m6A modification	Intermediary molecules	Interaction mode	References (PMID)
1	Liver fibrosis	High 5mc status of Socs3	YTHDF1	Stat3/HIF-1α	5mC-mediated Socs3 repression leads to Stat3 activation, which in turn activates HIF-1α. HIF-1α then activates YTHDF1.	36841889
2	Hepatocellular carcinoma	TET1	METTL3,METTL14,FXR1	SREBF2-AS1	METTL3 and METTL14 induce m6A modification, enhancing SREBF2-AS1 transcript stability and expression. SREBF2-AS1 recruits m6A readers FXR1 and TET1 to the SREBF2 promoter, causing its DNA demethylation and upregulating SREBF2 transcription.	38486042
3	Hypoxia-induced pulmonary artery endothelial cell pyroptosis	Methylation status of DRP1 promoter	YTHDC1	lncRNA-FENDRR	YTHDC1 bind to lncRNA-FENDRR to regulate its expression. lncRNA-FENDRR forms RNA-DNA triplexes in the promoter region of DRP1, leading to increased methylation levels at the DRP1 promoter, and reduced DRP1 transcription.	36284300
4	Diabetic retinopathy	1. MAP3K1 promoter hypomethylation; 2. Lactylation-related gene DNA methylation	FTO, METTL3; m6A modification of MAFG-AS1, CDK2 mRNA	MAFG-AS1, lncRNA SNHG7	1. METTL3-m6A stabilizes SNHG7, indirectly regulating DNA methylation regulators to affect MAP3K1 methylation; 2. DNA methylation-regulated histone lactylation upregulates FTO, which stabilizes MAFG-AS1 (*via* m6A demethylation) to alter local DNA methylation; 3. FTO-m6A modulates CDK2, which regulates DNA methyltransferase activity, forming bidirectional crosstalk.	40585189

In another case, METTL3/METTL14-mediated m6A modification enhances SREBF2-AS1 transcript stability and upregulates its expression. The m6A-modified SREBF2-AS1 binds and recruits m6A readers FXR1 and TET1 to the SREBF2 promoter, triggering DNA demethylation and promoting SREBF2 transcription. SREBF2 drives hepatocellular carcinoma initiation and progression by upregulating STARD4, modulating cell proliferation/migration, and regulating apoptosis-related pathways (Feng et al., 2023).

In hypoxia-induced pulmonary artery endothelial cell pyroptosis, YTHDC1 binds lncRNA-FENDRR to modulate its expression. FENDRR forms RNA-DNA triplexes at the DRP1 promoter, increasing DRP1 promoter methylation and reducing its transcription. DRP1 contributes to hypoxic pulmonary hypertension progression by regulating pyroptosis-related protein expression, modulating mitochondrial function, and affecting pulmonary vascular remodeling (Wang et al., 2022).

This intermediary-dependent relay pattern extends to diabetic retinopathy (DR) (Luo et al., 2025). In DR, curcumol activates the m6A demethylase FTO to reduce lncRNA MAFG-AS1 m6A modification, stabilizing MAFG-AS1 expression; concurrently, DR is accompanied by abnormal DNA methylation (*e.g*., MAP3K1 pathway promoter hypomethylation that promotes pathological progression). Mechanistically, FTO expression is regulated by histone lactylation—an intermediate that also modulates DNA methylation *via* epigenetic enzyme recruitment—while stabilized MAFG-AS1 may alter target gene DNA methylation *via* chromatin remodeling. Collectively, this forms a “curcumol-FTO/histone lactylation-MAFG-AS1-DNA methylation” relay that regulates DR progression.

Intermediary molecules act as bridges between m6A and DNA methylation, mediating their interaction *via* two core mechanisms: 1. Regulating the expression of epigenetic enzymes; 2. Modified intermediates binding to downstream gene promoters and recruiting regulatory proteins to control DNA methylation/demethylation. Drugs targeting these intermediates may disrupt downstream signaling activation and disease progression. For instance, in liver fibrosis (not ESCC, correcting tissue-disease correspondence), silencing Stat3 or HIF-1α inhibits YTHDF1 activation, thereby blocking the upregulation of collagen-related genes driving fibrosis progression. These intermediates exhibit great potential as cost-effective therapeutic targets with minimal side effects.

### Bidirectional regulation of DNAme and m6A

The DNAme-m6A and m6A-DNAme regulatory mechanisms discussed above are well-characterized unidirectional models. For DNAme and m6A, bidirectional regulatory mechanisms have rarely been reported to date, but one case has been relatively well characterized (Table 4)—a pattern that represents true “crosstalk” between the two modifications (Zhou, Tian & Qin, 2019). During tomato fruit ripening, the DNA demethylase SlDML2 plays a pivotal role in activating hundreds of genes associated with ripening. The m6A demethylase SlALKBH2 directly binds to SlDML2 mRNA, as demonstrated by RNA immunoprecipitation (RIP) assays, and mediates m6A demethylation within the 3′ untranslated region (3′UTR) of SlDML2 transcripts. This demethylation event alleviates the inhibitory effect of m6A modification on mRNA stability. Consistent with this, actinomycin D-based transcription inhibition assays confirmed a significant reduction in the degradation rate of SlDML2 mRNA, supporting the conclusion that SlALKBH2 enhances SlDML2 expression post-transcriptionally by stabilizing its mRNA.

**Table 4:** Bilateral regulation, co-regulation and co-expression of DNA methylation and m6A modification.

No.	Diseases	DNA methylation	m6A modification	Interaction mode	References (PMID)
1	Tomato fruit ripening	SlDML2	SlALKBH2	SlDML2 regulate SIALKBH2 and SIALKBH reciprocally regualted SIDML2.	31387610
2	Ossification of ligamentum flavum	DNMT1	FTO	SOCS3 expression exhibits a negative correlation with DNMT1 and a positive correlation with FTO.	35712246
3	Kupffer cell activation	DNMT1, DNMT3A, DNMT3B, TET2	METTL3, METTL14, IGF2BP1	5mC promotes PCK2 expression at the transcriptional level and that m6A promotes PCK2 expression at the post-transcriptional level.	39337381
4	Pregnancy-related tissues (bovine/ovine caruncle, bovine mammary)	1. Promoter 5mC (reduced in GREM1); 2. Genomic 5mC (on ARSI, GCM1)	1.GREM1 gene-body m6A hypermethylation; 2. ARSI/GCM1 transcript m6A	1.GREM1 promoter 5mC hypomethylation (reduces transcriptional repression) synergizes with gene-body m6A hypermethylation (enhances transcript stability), jointly upregulating GREM1 expression; 2. ARSI/GCM1 are dually modified by 5mC (genomic) and m6A (transcript, mainly gene-body), which co-regulate 22 downstream pregnancy-related genes.	40405312
5	Hepatic lipid deposition in laying hens	1. FASN/SCD promoter 5mC hypermethylation; 2. CPT1A promoter 5mC hypomethylation	SREBP1 mRNA 3′UTR m6A hypermethylation	Dietary betaine mediates DNA 5mC and m6A co-regulation of fatty acid metabolic genes: 1. 5mC hypermethylation of FASN/SCD promoters suppresses their expression (inhibits fatty acid synthesis); 2. 5mC hypomethylation of CPT1A promoter enhances its expression (promotes fatty acid β-oxidation); 3. m6A hypermethylation of SREBP1 mRNA 3′UTR reduces its transcript stability, downregulating SREBP1 expression—collectively reducing hepatic triglyceride deposition	40592294
6	H_2_O_2_-induced premature & replicative senescence (human lung fibroblasts)	1. Replicative: 1,810 hyper-/251 hypo-DMRs; 2. Premature: 1,090 hyper-/308 hypo-DMRs	1. Replicative: 691 hyper-/673 hypo-m6A peaks; 2. Premature: 1,042 hyper-/1,460 hypo-m6A peaks	5mC (regulates CDC45/TPX2/UBE2T) and m6A (regulates ASPM/CENPF) synergize to promote senescence (cell cycle arrest, SASP upregulation)	40521894
7	*Coprinopsis cinerea* development	CcTet	CcTet	CcTet works on both 5mC and 6mA demethylation.	38324471
8	Cancer	TET1 (and other DNA methylation genes in epigenetic module, EME)	YTHDC1 (and other m6A genes in EME)	These two types of genes are highly correlated.	32188475
9	Hepatocellular carcinoma	DNMT1 (and other DNA methylation genes in epigenetic module, EME)	METTL3 (and other m6A genes in EME)	The two types of analyses are highly correlated, interact with each other, and work synergistically	36627693
10	Neuroticism, depression, and narcolepsy, asthma, lung disease, coronary artery disease	61,775 DNA methylation-to-m6A regulatory loci (*e.g*., CBFA2T3-RBM15, DNMT1-METTL3, DNMT1-ALKBH1, POLR2A-METTL3/METTL14/WTAP)	4,733 m6A-to-DNA methylation loci (*e.g*., ATXN2-POLR2A, PTBP1-POLR2A, ATXN2CTCF, YTHDF2-POLR2A)	These regulatory pairs should influence each other	38981476
11	Pregnancy-related tissues (bovine/ovine caruncle, mammary, spleen)	TET1, DNMTs (regulate 5mC in gene-body (DB) and promoter (DP))	METTL3, METTL14, YTHDFs (regulate m6A in gene-body (RB) and promoter (RP))	1. DNAme-m6A co-expression: RM overlaps more with GE than DM, RNA unmethylated genes (RUM) associates with non-expressed genes (GNE); 2. 5mC (DB-DP) and m6A (RB-RP) have positive intra-regional interactions, cross-type (5mC DB-m6A RP, 5mC DP-m6A RB) interactions negative; 3. 1,062 cross-species pairs, 15 core genes (*e.g*., ARSI, GCM1) with RB-GE positive correlations.	40405312

**Note**:

1. Bilateral Regulation; 2–7: Co-regulation; 8–11: Co-expression.

Notably, SlALKBH2 transcription is also regulated by DNA methylation: SlDML2 targets a differentially methylated region (DMR) in the SlALKBH2 promoter (979–1,080 bp upstream of the start codon). Dual-luciferase reporter assays showed that SlDML2 enhances SlALKBH2 promoter activity, and bisulfite sequencing revealed that SlDML2 reduces 5mC levels in this DMR, thereby relieving transcriptional repression of SlALKBH2. This forms a functional closed positive feedback loop: SlALKBH2 stabilizes SlDML2 mRNA *via* m6A demethylation; increased SlDML2 protein levels further promote DNA demethylation of SlALKBH2 (and other ripening-related genes), thereby accelerating fruit ripening. Conversely, in the DNA-hypermethylated Cnr mutant or CRISPR/Cas9-edited slalkbh2 mutants, SlALKBH2 expression declines, SlDML2 mRNA m6A levels increase (validated by m6A-IP-qPCR), and fruit ripening is delayed. In such regulatory pairs, targeting either molecule disrupts the feedback loop, thus modulating physiological processes (*e.g*., fruit ripening) or disease initiation.

### Co-regulation of DNAme and m6A on the same targets

In addition to the regulation between DNAme and m6A, researchers have also identified molecular pairs that may act synergistically on the same target (Table 4). For instance, SOCS3 is a key node gene in the inflammatory process of ligamentum flavum ossification (OLF). SOCS3 expression exhibits a negative correlation with DNMT1 and a positive correlation with FTO (Zhang et al., 2022). DNMT1 and FTO may thus cooperatively regulate the SOCS3 gene to modulate OLF progression. In hepatitis, abnormal PCK2 expression triggers excessive activation of inflammatory responses, promoting liver inflammation progression. The key gene PCK2 is co-regulated by 5mC (transcriptional level) and m6A (post-transcriptional level): 5mC and m6A may serve as key targets for PCK2 modulation, thus providing a novel therapeutic strategy for liver inflammation. This co-regulatory pattern is widespread across species and biological processes, with a conserved logic: 5mC and m6A synergistically modulate target gene expression to support tissue-specific or process-specific functions.

Such co-regulation is also widespread across diverse species and biological processes. In bovine and ovine cross-species studies, 5mC and m6A jointly regulate tissue-specific functional genes: in pregnancy-critical caruncle tissue, ARSI and GCM1 are dually modified by genomic 5mC and transcriptomic m6A to control 22 pregnancy-related downstream genes, while in pregnant bovine mammary tissue, GREM1 (a placental development regulator) shows elevated gene-body m6A and reduced promoter 5mC, synergistically boosting its expression to support pregnancy (Xie et al., 2025). In laying hens, betaine mediates 5mC and m6A crosstalk to target hepatic lipid metabolism genes—5mC is increased on FASN/SCD promoters (suppressing transcription) and decreased on CPT1A promoter (enhancing expression), while m6A is elevated in SREBP1 mRNA 3′UTR (accelerating degradation) (Yang et al., 2025). In human embryonic lung fibroblasts, 5mC and m6A co-target cell cycle-related genes (*e.g*., m6A modifies ASPM, 5mC modifies CDC45) and co-enrich in senescence pathways, synergistically driving cell cycle arrest and senescence (Zhu et al., 2025).

Another interesting case of 5mC and m6A interaction was observed in *Coprinopsis cinerea*. There exists an atypical bifunctional dioxygenase CcTet, which is effective in demethylating both 5mC and 6mA, achieving “One Molecule, Double Agent” (Zhang et al., 2024a). The DNA methylation modifications 5mC and 6mA, play a crucial role in the regulation of gene expression, and the bifunctional enzyme CcTet in *Coprinopsis cinerea* can act on both of these modifications, making it a key molecule in the study of gene regulatory networks.

For targets dually regulated by DNA methylation and m6A, targeted therapeutic agents could be developed to exert coordinated epigenetic control over downstream genes, thereby enhancing clinical efficacy.

### Co-expression and potential interactions

Beyond experimentally validated interactions between DNAme and m6A, several studies have leveraged bioinformatics analyses to identify their co-expression patterns and potential cooperative effects (Table 4). A highly cited study conducted a systematic pan-cancer genomic analysis by mapping the molecular correlations between m6A and 5mC regulators in approximately 11,000 subjects with various types of cancer. Key findings revealed conserved patterns among regulators: most m6A and 5mC regulators exhibited similar expression levels across 33 cancer types, shared comparable mutation frequencies, and showed significant co-occurrence of genetic alterations. Additionally, regulators within the same class displayed correlated expression patterns, and m6A/5mC regulator expression was highly correlated—with frequent interactions in protein-protein interaction (PPI) networks (Chen et al., 2020). An m6A/5mC epigenetic module eigengene (EME) model has also been established to predict patient survival in most cancer types. EME is a composite signature integrating hub genes from m6A and 5mC regulatory networks, reflecting the overall epigenetic state. Across cancer types, the number of m6A hub genes was highly correlated with that of 5mC hub genes—a pattern likely driven by crosstalk between 5mC regulators, laying the foundation for EME construction. EME expression correlates closely with tumor proliferation/invasion, the tumor-immune-stromal microenvironment, and cancer patient prognosis, thus serving as a reference for clinical treatment decisions. Subsequently, another research group developed a similar m6A/5mC EME scoring system for liver cancer, further confirming the critical role of m6A-DNAme crosstalk in cancer pathogenesis (Tian et al., 2023).

Quantitative trait loci (QTLs) have further been employed as genetic tools to delineate m6A-DNAme crosstalk in diverse human diseases (*e.g*., neuroticism, depression, narcolepsy, asthma, lung disease, coronary artery disease) (Li et al., 2024a). These analyses identified 4,733 m6A-to-DNAme and 61,775 DNAme-to-m6A regulatory loci, which serve as a resource for comprehensively interpreting epigenetic crosstalk in human diseases (Li et al., 2024a). Mechanistically, the study found m6A regulates DNA methylation site distribution: DNA methylation sites are enriched near enhancers and transcription start sites (TSS). For example, in lung tissue, m6A modulates DNA methylation sites associated with TNFSF13 (*e.g*., cg10788408), altering chromatin state to facilitate transcription factor binding at gene regulatory regions—ultimately driving abnormal expression of disease-related genes and disease pathogenesis. Conversely, DNA methylation influences m6A site distribution: m6A sites are enriched in active genomic regions, depleted in repressive regions, and concentrated near transcription termination sites (TTS) (*e.g*., ACBD3-AS1-associated sites in lung tissue). This distribution pattern modulates mRNA stability and translation efficiency, altering gene expression to contribute to disease onset. Notably, this study relied primarily on large-scale bioinformatics analyses, with limited experimental validation for m6A-DNAme interactions; the specific mechanisms underlying their crosstalk require further experimental confirmation (Li et al., 2024a).

Notably, such co-expression patterns and functional interplay are not limited in cancer or other human diseases, but also observed in normal physiological processes across species. A multi-omics analysis of bovine and ovine tissues (caruncle, mammary gland, spleen) (Xie et al., 2025) revealed that m6A-modified genes (RM) overlapped more with actively expressed genes (GE) than 5mC-modified genes (DM); RNA unmethylated genes (RUM) and non-expressed genes (GNE) also showed strong associations. Linear regression further demonstrated positive intra-regional interactions for same-type modifications (*e.g*., gene-body 5mC (DB) *vs*. promoter 5mC (DP), gene-body m6A (RB) *vs*. promoter m6A (RP)) but negative cross-type interactions between gene-body and promoter regions (*e.g*., DB *vs*. RP, RB *vs*. DP). Moreover, 1,062 of these interaction pairs were conserved across bovines and ovines, with 15 core genes (*e.g*., ARSI, GCM1) maintaining consistent RB-GE positive correlations, underscoring the evolutionary conservation and functional relevance of these co-expression patterns.

Collectively, these co-expressed DNAme and m6A regulators are predicted to mediate functional crosstalk, rendering them compelling candidates for subsequent mechanistic investigation and therapeutic targeting.

## Discussion

### Trends and implications of DNAme–m6A interaction research

DNAme and m6A regulations have been studied independently for decades: DNAme research, traceable to 1963, now exceeds 100,000 publications in Web of Science Core Collection (WOSCC) and saw accelerated growth post-2000; m6A research, initiated in 1976, but progressed slowly until a surge after 2017, with ~9,000 WOSCC publications to date. In stark contrast, investigations into DNAme–m6A crosstalk only emerged in 2019, and merely 29 original articles addressing direct interactions were published by 2025—underscoring that this field is still in its infancy and holds considerable untapped potential.

From the bibliometric perspective (Fig. 3A), the temporal and modal distribution of publications reveals critical insights: 1. Modes with concentrated activity (*e.g*., mode 1 in 2021, mode 5 in 2025) one possibility is that they likely reflect “hotspots” driven by technological advancements (*e.g*., new tools for profiling DNAme and m6A simultaneously) or breakthroughs in understanding downstream functional consequences; 2. The scarcity of publications in modes 3 (indirect interplay *via* intermediates) and 4 (direct bilateral regulation) suggests these mechanisms are either more challenging to characterize experimentally or genuinely less prevalent—presenting opportunities for targeted method development or hypothesis-driven exploration.

Geographically, China demonstrates dominant and comprehensive contributions to DNAme–m6A crosstalk research across all six interaction modes. Not only does China have publications in every mode, but it also sustains consistent high-impact output over years (2019–2025). In contrast, the USA contributes moderately to specific modes (*e.g*., mode 1, mode 2) with relatively limited citation scale, while Denmark’s participation is restricted mainly to mode 1. This disparity reflects China’s robust research focus—potentially driven by strategic investments in epitranscriptomics/epigenomics, abundant talent pools, or synergies between clinical and basic science initiatives—whereas the USA and Denmark exhibit narrower research priorities or resource allocation in this field. Such geographic differences may shape international collaboration patterns and steer the global trajectory of the field. We propose that these disparities could be addressed through targeted collaborative initiatives to balance global research efforts.

Notably, mode 4 (direct bilateral regulation between DNAme and m6A) garners the highest single-mode citation count (221 citations) despite only one published study, which underscores intense research interest in true reciprocal DNAme–m6A regulatory mechanisms and highlights the need for expanded investigation into such interaction modalities in the future. Collectively, these bibliometric trends not only map the current state of DNAme–m6A interaction research but also pinpoint gaps (*e.g*., understudied modes, geographic imbalances) and opportunities (*e.g*., leveraging technological innovations to dissect uncharacterized mechanisms) that will guide future investigations.

### The interplay of DNAme and m6A

This article presents a comprehensive overview of the interplay between DNAme and m6A. It offers a systematic classification of their interactions, providing valuable research directions and conceptual frameworks for future investigations into the crosstalk between DNA methylation and m6A. Furthermore, the distribution of studies across different categories reflects current research priorities and hotspots. For instance, the category “DNAme modulates m6A” comprises the largest number of studies (nine studies), whereas “direct bilateral regulation between DNAme and m6A” is the least explored (only 1 study). This disparity highlights promising yet underexplored areas that warrant further investigation, which may yield novel and insightful findings.

Understanding the interaction between DNAme and m6A is of significant importance for advancing the field of epigenetics. Gene regulation is a complex, multifaceted, and interconnected process within biological organisms. One of the significant challenges in translating rapid advancements in basic medical research into clinical applications is that, within the intricate milieu of biological organisms, the function of individual molecules can easily be disrupted and obscured. Therefore, studying the overall regulation pattern of genes can help clarify the pathogenic mechanisms in depth and may effectively enhance the efficiency of gene therapy.

**Figure 5:**
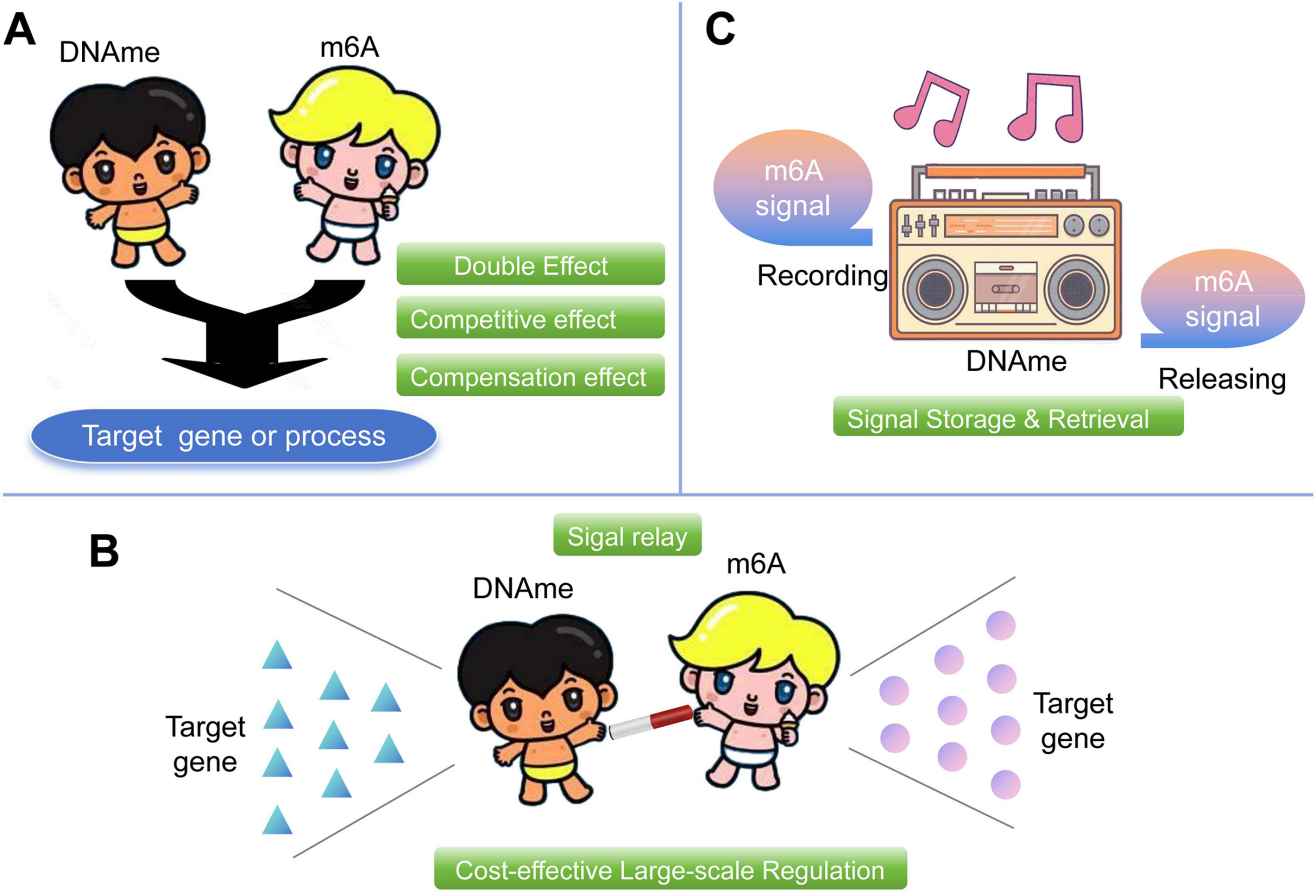
Potential functional implications of the crosstalk between DNA methylation and m6A modification. (A) Double enhanced/Competitive/Compensation effect on the same target gene or biological process. (B) Signal relay and cost-effective large-scale regulation. (C) Signal storage and rapid release.

DNAme and the emerging m6A modification exert control at the transcriptional and post-transcriptional levels respectively. If there is a joint action between the two (Fig. 5A), their regulatory scope and effects would be significant and extensive which deserve in-depth research. For instance, in hepatitis, the abnormal expression of PCK2 may lead to excessive activation of inflammatory responses, thereby promoting the progression of liver inflammation. The key gene, PCK2, can be co-regulated by 5mC and m6A at both the transcriptional and post-transcriptional levels (Zhao et al., 2024). Therefore, we propose that dual-target therapy using 5mC and m6A may have better clinical outcomes.

Notably, the interaction between DNAme and m6A is not limited to collaborative regulation of the same gene or biological process; it also involves functional competition and compensatory effects (Fig. 5A) that enhance the robustness of the regulatory network. A study on pregnant cattle and sheep tissues found that the same type of methylation (either DNAme or m6A) shows positive synergy between gene bodies and promoters, ensuring consistent regulatory directions across genomic regions (Xie et al., 2025). In contrast, cross-type methylation between gene bodies and promoters presents negative antagonism: when one type of methylation (*e.g*., DNAme in promoters) inhibits transcription, the other (*e.g*., m6A in gene bodies) can be upregulated to compensate—for example, by enhancing mRNA stability—to maintain stable gene expression, as seen in pregnancy-related genes like GREM1 and PODXL (Xie et al., 2025). This competition and compensation prevent extreme fluctuations in gene expression caused by single-modification abnormalities, safeguarding critical biological processes (such as placental development). It not only deepens our understanding of multi-layered epigenetic-epitranscriptomic regulation but also suggests that targeting key nodes in this network could enable more precise, mild biological regulation, laying a foundation for developing efficient, low-side-effect targets. We propose that identifying these key nodes could be a priority for translational research.

On the other hand, in large-scale bioinformatics analyses, the expression of m6A and 5mC regulatory factors was highly correlated and frequently interacted within protein-protein interaction networks, indicating a close relationship between these two. Why do they interact frequently? One possibility is that they are both important regulatory molecules in epigenetic and epitranscriptomic field, and their regulation can lead to the activation or suppression of a large number of downstream genes. Therefore, DNAme and m6A can act as hub regulatory genes that exert leveraged control effects. For example, during the grafting process of apples, the downregulation of the m6A gene MdMTA can induce global hypomethylation of m6A and significantly activate the expression level of the DNA demethylase gene MdROS1, thereby participating in the regulation of stress-responsive genes during the apple grafting process (Xu & He, 2023). Here, the DNAme gene plays a notable role as a “signal relay baton”. Moreover, this “signal-relay baton” regulates a wide range of genes and has a potentially cost-effective role in biological regulatory systems (Fig. 5B).

Furthermore, DNAme is a relatively stable and heritable pre-transcriptional control, whereas the m6A modification is a relatively dynamic and reversible post-transcriptional control. We hypothesize that their interaction orchestrates a sophisticated mechanism for epigenetic memory in diseases like asthma. Specifically, DNAme may encode long-term, potentially heritable “memories” of disease exacerbations, progressively lowering the threshold for subsequent attacks. Conversely, m6A modification could act as a rapid, tunable regulator, critically involved in both the initial “writing” of this DNAme-based memory during inflammatory episodes and its subsequent “reading” or functional output during symptom manifestation (Fig. 5C). Targeting this crosstalk—either by disrupting m6A-mediated memory inscription to prevent pathological recording or by blocking m6A-dependent recall of established DNAme patterns—may hold therapeutic promise for halting disease progression and its potential intergenerational transmission. Consequently, investigating whether these modifications apply distinctly to different disease types (*e.g*., genetic, chronic, acute) and dissecting the dynamics of their interaction, initiation, or dormancy across disease stages are crucial research imperatives and this requires validation through further experimental and clinical studies.

Notwithstanding the systematic classification of DNAme–m6A interactions herein, current studies still harbor notable discrepancies that warrant attention. In Mode 1 (DNAme modulates m6A, Table 4), for instance, ALKBH5 expression is upregulated by hypermethylation of its promoter in lung cancer (Zhang et al., 2021) (Table 1, No. 6)—*via* blocking the transcriptional repressor CTCF—but by hypomethylation of the same promoter in esophageal squamous cell carcinoma (Wu et al., 2020) (Table 1, No. 7), reflecting divergent regulatory outcomes driven by DNAme’s target elements (repressor *vs*. basal transcriptional sites). Similarly, in Mode 2 (m6A regulates DNAme, Table 2), METTL3 promotes DNAme elevation *via* upregulating DNMT1 in non-small cell lung cancer (Li et al., 2024b) (Table 2, No. 1) yet drives DNAme reduction by recruiting TET1 in esophageal squamous cell carcinoma (Deng, Zhang & Su, 2022) (Table 2, No. 3), a contradiction rooted in the distinct DNAme regulators (methyltransferase *vs*. demethylase) targeted by m6A. Even the well-observed DNMT1-METTL3 pair exhibits conflicting relationships: DNMT1 negatively regulates METTL3 in pancreatic cancer (Zhang et al., 2019) (Mode 1, Table 1, No. 2) but shows positive co-expression and synergy with METTL3 in hepatocellular carcinoma (Tian et al., 2023) (Mode 6, Table 4, No. 9), highlighting unaddressed context dependencies (disease-specific regulatory networks). These discrepancies, far from invalidating current findings, underscore the need for future studies to explicitly characterize key variables—such as DNAme’s regulatory elements, m6A reader proteins, and disease subtypes—to resolve ambiguities and advance a more unified understanding of DNAme–m6A crosstalk.

## Conclusion and future perspectives

This article provides a systematic bibliometric analysis of the burgeoning field of crosstalk between DNAme and m6A. Our findings delineate a complex and multifaceted regulatory network, encompassing direct (DNAme-m6A, m6A-DNAme) and indirect (*via* intermediate molecules) mechanisms, alongside bidirectional regulation and co-expression patterns. This intricate interplay between the well-established DNAme and the emerging star of m6A underscores the multi-layered complexity of epigenetic and epitranscriptomic regulation.

This study has several limitations to consider. First, relying solely on the Web of Science Core Collection (WOSCC) may have omitted relevant studies from other databases (*e.g*., PubMed, Scopus). However, this is a common bibliometric practice to prioritize data quality and high-impact journal coverage; we also used a broad search strategy and reference screening to mitigate omissions, with future multi-database studies able to expand these findings. Second, the field of DNAme–m6A interaction research remains exploratory, with only 29 studies directly investigating their crosstalk included. This small sample size restricts conclusion breadth—for example, limiting validation of the six proposed interaction modes across contexts—and findings should be interpreted cautiously until more data accumulate. Third, the geographic distribution of included studies (83% from China, 11% from the US, 1 from Denmark, no other countries) reflects the field’s current landscape, not our search bias; our WOSCC screening confirmed no additional eligible studies from other regions exist. This limited global participation highlights that the field is predominantly driven by Chinese and US teams, an observation that can guide future global collaboration to diversify research. Fourth, current evidence is dominated by correlative data (*e.g*., co-expression of DNMTs and METTL3, concurrent DNAme/m6A changes) rather than causal validation (*e.g*., CRISPR-mediated knockout), limiting definitive conclusions on crosstalk directionality and mechanisms. Fifth, there is clear context bias: while studies cover human cancers (pancreatic, lung, esophageal squamous cell, hepatocellular carcinoma), diseases (cardiac hypertrophy, diabetic retinopathy, asthma), physiological processes (porcine embryo development, tomato fruit ripening), and non-mammalian systems (*Coprinopsis cinerea*), over half focus on human cancers. Scarce research explores regulatory patterns across distinct contexts, hindering understanding of DNAme–m6A interplay’s universal *vs*. context-specific roles.

To advance understanding of DNAme–m6A crosstalk, future research should combine scope expansion with mechanistic deepening by leveraging targeted experimental strategies: integrated multi-omics profiling—combining whole-genome bisulfite sequencing, m6A RNA immunoprecipitation sequencing, transcriptomics, and proteomics—will map co-occurring DNAme and m6A modifications at high resolution to link epigenetic-epitranscriptomic patterns to functional outcomes; single-cell resolution techniques (*e.g*., single-cell bisulfite sequencing paired with single-cell m6A sequencing) will resolve cell-type-specific interaction modes, overcoming the averaging effects of bulk analyses in heterogeneous systems like tumor microenvironments or developing tissues; and CRISPR-based perturbation studies—including targeted knockout/activation of key regulators (*e.g*., DNMTs, METTL3) and rescue experiments—will validate causal relationships to clarify the directionality of crosstalk and identify critical regulatory nodes. Additionally, expanding research beyond cancer to understudied contexts (*e.g*., autoimmune diseases, metabolic disorders, non-mammalian systems) will reveal universal *vs*. context-specific interaction rules, collectively transforming descriptive observations into actionable mechanistic insights that enable therapeutic targeting of DNAme–m6A crosstalk in disease.

## Supplemental Information

10.7717/peerj.20654/supp-1 Supplemental Information 1 The schematic illustration of the six main interplay mechanism of DNA methylation and m6A modification. DNA methylation(DNAme)-m6A modification regulation; m6A-DNAme regulation; The interplay of DNAme with m6A *via* intermediate molecules; Direct bilateral regulation of DNAme and m6A molecules; Co-regulation of DNAme and m6A on the same target; Co-expression and potential interactions of these two molecules.

10.7717/peerj.20654/supp-2 Supplemental Information 2 The detailed information of 29 articles for bibliometric analysis.

## Additional Information and Declarations

### Data Availability

The following information was supplied regarding data availability:

This is a literature review.
