# Supplementary figures and images for: The orchestrated interplay between DNA methylation and N6-methyladenosine modification: status quo and future perspectives
Source: PeerJ. 2026 Jan 27;14:e20654. doi: 10.7717/peerj.20654 (PMC12857562; doi:10.7717/peerj.20654)

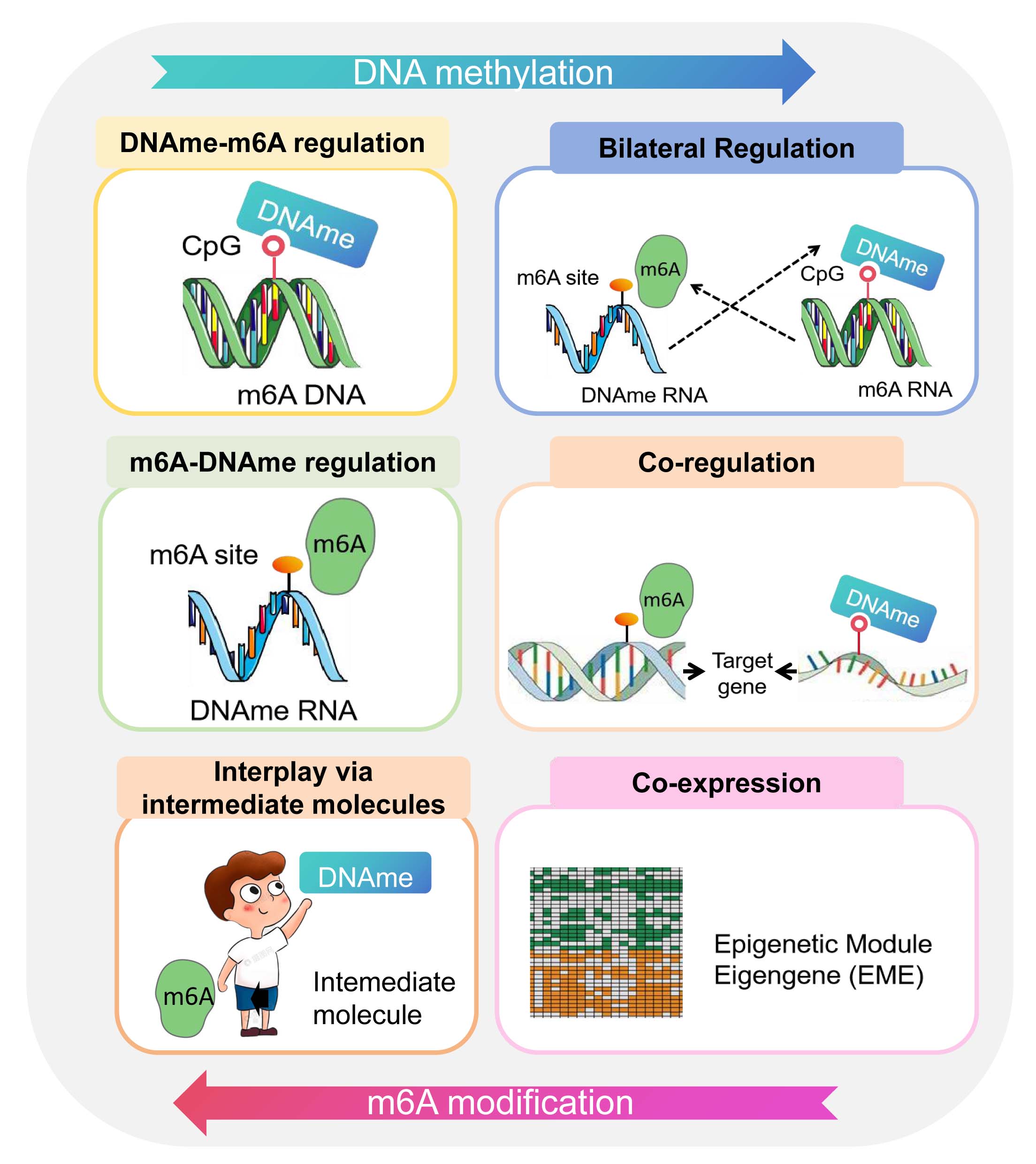

Supplement: Supplemental Information 1 — DNA methylation(DNAme)-m6A modification regulation; m6A-DNAme regulation; The interplay of DNAme with m6A via intermediate molecules; Direct bilateral regulation of DNAme and m6A molecules; Co-regulation of DNAme and m6A on the same target; Co-expression and potential interactions of these two molecules. [file peerj-14-20654-s001.jpg]
