# Supplementary material for: The orchestrated interplay between DNA methylation and N6-methyladenosine modification: status quo and future perspectives
Source: PeerJ. 2026 Jan 27;14:e20654. doi: 10.7717/peerj.20654 (PMC12857562; doi:10.7717/peerj.20654)
Supplement: Supplemental Information 2 [file peerj-14-20654-s002.docx]

| **Table S1.The detailed information of 29 articles for bibliometric analysis.** | | | |
| --- | --- | --- | --- |
| Interaction Mode Type | Article Title | Source Journal | Pubmed Id |
| 1 | Excessive miR-25-3p maturation via N6-methyladenosine stimulated by cigarette smoke promotes pancreatic cancer progression | NATURE COMMUNICATIONS | 31015415 |
| 1 | A Novel Micropeptide Encoded by Y-Linked LINC00278 Links Cigarette Smoking and AR Signaling in Male Esophageal Squamous Cell Carcinoma | CANCER RESEARCH | 32169859 |
| 1 | Suppression of m6A mRNA modification by DNA hypermethylated ALKBH5 aggravates the oncological behavior of KRAS mutation/LKB1 loss lung cancer | CELL DEATH & DISEASE | 34016959 |
| 1 | DNA methylation of FTO promotes renal inflammation by enhancing m6A of PPAR-α in alcohol-induced kidney injury | PHARMACOLOGICAL RESEARCH | 33157234 |
| 1 | A comprehensive epigenome atlas reveals DNA methylation regulating skeletal muscle development | NUCLEIC ACIDS RESEARCH | 33434283 |
| 1 | Maternal obesity increases DNA methylation and decreases RNA methylation in the human placenta | REPRODUCTIVE TOXICOLOGY | 34890771 |
| 1 | Bioinformatics analysis identifies potential m6A hub genes in the pathogenesis of intracerebral hemorrhage | JOURNAL OF NEUROIMMUNOLOGY | 37907028 |
| 1 | Reduced DNMT1 levels induce cell apoptosis via upregulation of METTL3 in cardiac hypertrophy | HELIYON | 38314261 |
| 1 | DNA methylation regulates RNA m6A modification through transcription factor SP1 during the development of porcine somatic cell nuclear transfer embryos | CELL PROLIFERATION | 38095020 |
| 1 | Coordinated transcriptional and post-transcriptional epigenetic regulation during skeletal muscle development and growth in pigs | JOURNAL OF ANIMAL SCIENCE AND BIOTECHNOLOGY | 36457054 |
| 2 | RNA m6A regulates transcription via DNA demethylation and chromatin accessibility | NATURE GENETICS | 36071173 |
| 2 | Global hypermethylation of the N6-methyladenosine RNA modification associated with apple heterografting | PLANT PHYSIOLOGY | 37648253 |
| 2 | Crosstalk between RNA m6A and DNA methylation regulates transposable element chromatin activation and cell fate in human pluripotent stem cells | NATURE GENETICS | 37474847 |
| 2 | METTL3-mediated m6A methylation of DNMT1 promotes the progression of non-small cell lung cancer by regulating the DNA methylation of FOXO3a | HELIYON | 38586389 |
| 2 | RNA m6A modification facilitates DNA methylation during maize kernel development | PLANT PHYSIOLOGY | 37995374 |
| 3 | LncRNA FENDRR with m6A RNA methylation regulates hypoxia-induced pulmonary artery endothelial cell pyroptosis by mediating DRP1 DNA methylation | MOLECULAR MEDICINE | 36284300 |
| 3 | DNA 5mC and RNA m6A modification successively facilitates the initiation and perpetuation stages of HSC activation in liver fibrosis progression | CELL DEATH AND DIFFERENTIATION | 36841889 |
| 3 | METTL3 and METTL14-mediated N6-methyladenosine modification of SREBF2-AS1 facilitates hepatocellular carcinoma progression and sorafenib resistance through DNA demethylation of SREBF2 | SCIENTIFIC REPORTS | 38486042 |
| 3 | Curcumol targets the FTO/MAFG-AS1 axis to alleviate diabetic retinopathy via epigenetic remodeling and nanodelivery-based microenvironment modulation | WORLD JOURNAL OF DIABETES | 40585189 |
| 4 | RNA methylomes reveal the m6A-mediated regulation of DNA demethylase gene SlDML2 in tomato fruit ripening | GENOME BIOLOGY | 31387610 |
| 5 | Deciphering Obesity-Related Gene Clusters Unearths SOCS3 Immune Infiltrates and 5mC/m6A Modifiers in Ossification of Ligamentum Flavum Pathogenesis | FRONTIERS IN ENDOCRINOLOGY | 35712246 |
| 5 | DNA 5mC and RNA m6A Collaborate to Upregulate Phosphoenolpyruvate Carboxykinase 2 for Kupffer Cell Activation | INTERNATIONAL JOURNAL OF MOLECULAR SCIENCES | 39337381 |
| 5 | Molecular basis of an atypical dsDNA 5mC/6mA bifunctional dioxygenase CcTet from Coprinopsis cinerea in catalyzing dsDNA 5mC demethylation | NUCLEIC ACIDS RESEARCH | 38324471 |
| 5 | Betaine decreases hepatic lipid deposition through DNA 5 mC and RNA m6A methylation-mediated regulation of fatty acid metabolic genes expression in laying hens | POULTRY SCIENCE | 40592294 |
| 5 | Global m6A RNA and whole 5mC DNA methylation specifically contribute to cell replicative and premature senescence induced by extrinsic oxidative stress | MSYSTEMS | 40521894 |
| 5/6 | Analyzing the relationship of RNA and DNA methylation with gene expression | GENOME BIOLOGY | 40405312 |
| 6 | Identification of cross-talk between m6A and 5mC regulators associated with onco-immunogenic features and prognosis across 33 cancer types | JOURNAL OF HEMATOLOGY & ONCOLOGY | 32188475 |
| 6 | Crosstalk between 5-methylcytosine and N6-methyladenosine machinery defines disease progression, therapeutic response and pharmacogenomic landscape in hepatocellular carcinoma | MOLECULAR CANCER | 36627693 |
| 6 | Crosstalk between epitranscriptomic and epigenomic modifications and its implication in human diseases. | Cell genomics | 38981476 |
